# Risk of bias assessment in preclinical literature using natural language processing

**DOI:** 10.1002/jrsm.1533

**Published:** 2021-11-05

**Authors:** Qianying Wang, Jing Liao, Mirella Lapata, Malcolm Macleod

**Affiliations:** ^1^ Centre for Clinical Brain Sciences University of Edinburgh Edinburgh UK; ^2^ School of Informatics University of Edinburgh Edinburgh UK

**Keywords:** automatic assessment, natural language processing, preclinical research synthesis, risk of bias

## Abstract

We sought to apply natural language processing to the task of automatic risk of bias assessment in preclinical literature, which could speed the process of systematic review, provide information to guide research improvement activity, and support translation from preclinical to clinical research. We use 7840 full‐text publications describing animal experiments with yes/no annotations for five risk of bias items. We implement a series of models including baselines (support vector machine, logistic regression, random forest), neural models (convolutional neural network, recurrent neural network with attention, hierarchical neural network) and models using BERT with two strategies (document chunk pooling and sentence extraction). We tune hyperparameters to obtain the highest F1 scores for each risk of bias item on the validation set and compare evaluation results on the test set to our previous regular expression approach. The F1 scores of best models on test set are 82.0% for random allocation, 81.6% for blinded assessment of outcome, 82.6% for conflict of interests, 91.4% for compliance with animal welfare regulations and 46.6% for reporting animals excluded from analysis. Our models significantly outperform regular expressions for four risk of bias items. For random allocation, blinded assessment of outcome, conflict of interests and animal exclusions, neural models achieve good performance; for animal welfare regulations, BERT model with a sentence extraction strategy works better. Convolutional neural networks are the overall best models. The tool is publicly available which may contribute to the future monitoring of risk of bias reporting for research improvement activities.

**Highlights**

- Risk of bias assessment is critical to reduce translation issues from preclinical to clinical research.
- Animal intervention studies vary from clinical trials and automatic tools designed for risk of bias assessment in preclinical literature remain to be developed.
- We present and implement a series of natural language processing models for the classification of reporting of five preclinical risk of bias items.
- The open‐source tool provides the possibility for future research improvement activities.

## 1 BACKGROUND

Systematic review is a type of literature review that attempts to collate all empirical evidence relevant to a pre‐specified research question. It uses explicit and systematic methods to minimise bias and provide more reliable findings than narrative review.^1^ After the collection of research publications which meet pre‐specified inclusion criteria, a critical step is the reporting of strategies designed to reduce risks of bias in the included publications, which is central to the assessment of the reliability of the research findings.^2^ The current procedure for risk of bias assessment in literature is that it usually performed separately by two independent investigators, working with an adjudicator to resolve any disagreements. This is both time‐consuming and prone to error. As the number of publications describing experimental studies increases rapidly, it has become increasingly difficult for researchers to keep up to date with progress in their field and the findings of systematic reviews are weakened. Therefore, automation tools would accelerate this process and increase reliability. Such tools would also have been useful in evaluating the impact of measures designed to improve the quality and completeness of research reporting, for instance the NPG Quality in Publication (NPQIP) study,^3^ the Intervention to Improve Compliance with the ARRIVE guidelines (IICARus) studies,^4^ in future evaluation of reporting standards such as the Materials‐Design‐Analysis‐Reporting Minimum Standards Framework^5^ and in measuring the impact of institutional research improvement activities.^6^

Systematic reviewers have advocated the use of automated approaches to assist risk of bias assessment, using human effort and machine automation in mutually reinforcing ways.^7^ The development of machine learning and natural language processing (NLP), including neural models and transfer learning, provides opportunities to create robust tools for risk of bias assessment. For clinical trials, RobotReviewer trains support vector machines on 6610 full texts with pseudo labels derived from 1400 unique strings of bias domains from the Cochrane Database of Systematic Reviews, which achieves overall accuracy around 71%.^8^ Zhang et al. consider the supported sentence annotations of bias domains as ‘rationales’ and use them to train the convolutional neural networks (CNNs)^9^ which improves the performance by 5% compared to baseline models.^10^ Millard et al. apply logistic regressions on 1467 full‐text clinical reports for sentence and document classification separately and achieves an area under the ROC curve greater than 72% for randomisation sequence generation, allocation concealment and blinding.^11^ Menke et al. have reported the performance of a proprietary tool SciScore^12^ which trains the conditional random fields^13^ on 250 research articles with manually labelled entity mentions for random allocation and blinding. The training corpus is randomly selected from the PubMed Open Access articles, and the portion of clinical or preclinical publications is not clear.

Compared with clinical trials, animal studies are conducted in relatively small teams, are reported in a different style, have been shown to have lower reporting of strategies to reduce risks of bias,^14^ and are susceptible to different risks of bias.^15^ Hence, separate tools for risk of bias assessment in preclinical literature are necessary. Bahor et al. have previously reported the use of regular expressions with rule‐based string matching to recognise phrases related to risk of bias reporting in experimental animal studies, which requires many hand‐crafted term selections.^16^ NLP approaches may achieve more robust results in the preclinical literature compared with non‐learning algorithms.

Several reporting standards relevant to the design, conduct, analysis and reporting of animal studies have been suggested, including the ARRIVE guidelines^17^ and the Materials‐Design‐Analysis‐Reporting Minimum Standards Framework,^5^ and these each contain multiple domains relating to potential risk of bias. In 2012, a stakeholder group convened by the U.S. National Institute of Neurological Disorders and Stroke prioritised the importance of reporting randomisation and blinding, sample size estimation and data handling (including the reporting of data excluded from analysis). A 2020 systematic review identified 60 publications containing 58 recommendations, with the most frequently recommended being sample size calculation, blinding assessment of outcome, choice of statistical methods and randomised allocation to treatment group.^18^ In systematic reviews conducted by our group (see ‘Dataset’ below for description), the prevalence of reporting of allocation concealment and of sample size calculations is so low that we do not think there are sufficient positive instances to provide adequate training, and we believe that a judgement of the appropriateness of the statistical methods chosen is highly subjective. To the three remaining risks of bias (blinding, randomisation and reporting of data exclusions), we add two further items. We know that the reporting of conflicts of interests is substantially higher than other risks of bias, and want to test the performance of NLP models across a range of reporting prevalence; and regulatory agencies and others often express concerns that studies report compliance with animal welfare regulations, so we include this item.

## 2 MATERIALS AND METHODS

**FIGURE 1:**
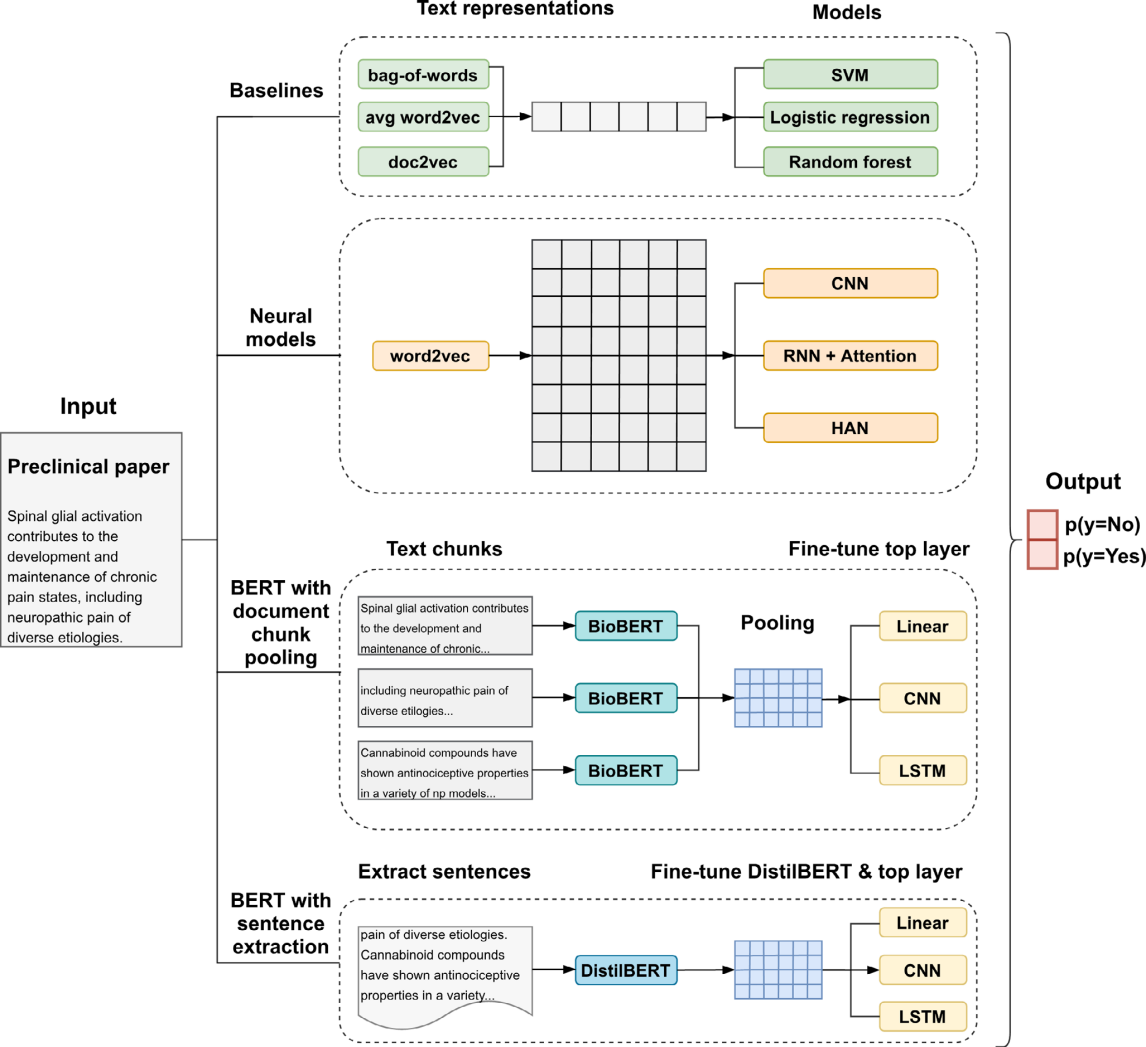
Overall methods of text representations and classification models being tested [Colour figure can be viewed at wileyonlinelibrary.com]

We consider the risk of bias assessment as a typical text classification task. A classification model cannot be trained from the plain text directly and we need to convert text information to analysable data. The core concept is to map each document to a matrix consisting of fixed‐dimension word vectors or embeddings,^19^ then train a classification model to map these numeric text representations to a binary risk of bias label (yes/no). For representation methods, we explore bag‐of‐words, word2vec,^20^ doc2vec^21^ and embeddings from BERT.^22^ For classification models, we implement baseline models (support vector machine, logistic regression, random forest), neural models (CNN, recurrent neural network (RNN) with attention, hierarchical neural network) and BERT models using two strategies, which are described in greater detail below. The different approaches are summarised in Figure 1, and training details are given in Supporting Information.

### 2.1 Dataset

We use a collection of full‐text publications which our group has annotated for risk of bias^23^ from two sources. Firstly, we use in‐house data from systematic reviews in (1) psychotic disorders^24^ (2386 publications); (2) chemotherapy‐induced peripheral neuropathy^25^ (1602 publications); and (3) several individually smaller systematic reviews in animal models of stroke, depression, hypertension, myocardial infarction and pain^22^ (2439 publications). Secondly, we have collected data in the context of observational (NPQIP,^3^ 751 publications) and experimental (IICARus,^4^ 662 publications) studies of interventions to improve the reporting of in vivo research. For the psychosis dataset, each manuscript had been evaluated by a single trained human reviewer and a regular expression approach,^16^ with disagreements reconciled by a second independent human reviewer. For all other datasets, each manuscript had undergone risk of bias annotation from two trained reviewers working independently, with differences reconciled by a third reviewer.

The risk of bias labels are at the document level (1 for reported, 0 for not reported) and we consider five risk of bias domains: (1) Random Allocation: animals are randomly allocated to treatment or control groups; (2) Blinded Assessment of Outcome: group identity is concealed from the scientist measuring the outcome; (3) Compliance with Animal Welfare Regulations: researchers report that they complied with relevant animal welfare regulations; (4) Conflict of Interests: authors report any relationship which might be perceived to introduce a potential conflict of interests, or the absence of such a relationship; and (5) Animal Exclusions: a statement of whether or not all animals, all data and all outcomes measured are accounted for and presented in the final analysis. The prevalence of reporting of each of these items, and some example sentences indicating the reporting for each risk of bias item are displayed in Table 1.

**TABLE 1:** Percentage of papers reporting each risk of bias item, and example sentences from full texts indicating the reporting

Risk of bias item	Reporting percentage	Positive example
Random allocation	27.5%	… a randomisation code is used to allocate animals to treatment group …
Blinded assessment of outcome	30.6%	… the midbrain sections from each animal were screened for … by a person unaware of the treatment condition of the animals …
Conflict of interests	78.0%	The authors declare that they have no competing interests
Animal welfare regulations	31.5%	… experiments were performed in accordance with protocols by the Institutional Animal Care and Use Committee at …
Animal exclusions	12.2%	… cases in which the lesion was assessed to involve less than <50% of the dopamine neurons, the animal was excluded from …

Publications were available in PDF format and we converted them to plain text using Xpdf (https://www.xpdfreader.com). We converted all text to lower case and used regular expressions to remove references, citations, URLs, digits, non‐ASCII characters and text which precedes the ‘Introduction’ section, because they are irrelevant to the risk of bias reporting. We used Stanford CoreNLP^26^ for word and sentence tokenization. After removing invalid records (for instance where text conversion failed), 7840 full‐text publications had annotations for random allocation, blinded assessment of outcome and animal exclusions, and 7089 had annotations for conflict of interests and animal welfare regulations. We combined publications from different source projects and randomly allocated them to training (80%), validation (10%) and test (10%) sets. Summary statistics of the dataset are shown in Table 2.

**TABLE 2:** Data statistics

	Samples for random allocation, blinded assessment of outcome and animal exclusions			Samples for conflict of interest and compliance with animal welfare regulations		
	Train	Valid	Test	Train	Valid	Test
No. documents	6272	784	784	5671	708	710
Avg No. tokens per document	4977	5112	5077	4947	5057	4964
Avg No. sentences per document	180	186	184	178	182	178
Avg No. tokens per sentence	28	28	28	28	28	28

*Note*: Samples for random allocation, blinded assessment of outcome and animal exclusions consist of 7840 records; samples for compliance of animal welfare regulations and conflict of interests consist of 7089 records.

### 2.2 Baselines

We explore three text representation methods in baseline models: (1) bag‐of‐words, (2) word2vec and (3) doc2vec. Bag‐of‐words (bow) uses word frequency within the document to represent its importance. Considering less important words with high frequency such as ‘the’ and ‘a’, TF‐IDF (term frequency‐inverse document frequency) weighting is applied, which normalises the word frequency in a document by multiplying a log‐scale of the inverse of the frequency of documents where the word occurred.^27^ Word2vec is a neural language model which learns to map words to continuous vectors. It can preserve the semantic relationship among words and can either be generated from the learning process jointly within the classification model or fine‐tuned on pre‐trained word vectors from other language tasks. As the preclinical literature belongs to the biomedical domain, we use the 200‐dimensional word vectors induced on a combination of PubMed and PubMedCentral texts with texts extracted from a recent English Wikipedia dump, using the skip‐gram model with a window size of 5.^28^ Doc2vec is an unsupervised method which learns to represent a document by a dense vector. There are two approaches for training the dense vector: Distributed Memory and Distributed Bag‐of‐Words, which are suggested to yield better performance when used together.^21^

We explore three baseline classifiers: support vector machine, logistic regression and random forest. Support vector machine and logistic regression are linear classifiers, which are trained to map the word embeddings to the target risk of bias label to minimise a hinge loss function and log loss function separately.^29^ Random forest is an ensemble‐based non‐parametric method which combines a number of decision trees trained on various sub‐samples.^30^

### 2.3 Neural models

We explore three neural models: CNN, a powerful model for text classification;^9^ RNN which is good at modelling sequential text data;^31^ and hierarchical attention network (HNN)^32^ which takes the hierarchical structure among word, sentence and document into consideration. The critical elements in the model architecture are described below and shown in Figures S1–S4.

#### 2.3.1 Convolutional Neural Network

We use the classic one‐layer CNN^9^ for document classification. The main characteristic of CNN is the convolutional layer where multiple filter windows (2D matrices) with different sizes are applied to filter out information. Let $x\left[i:j\right]$ denote the matrix extracted from row i to row j of the document matrix. For one document matrix $x\;\in \;{\mathbb{R}}^{s\times d}$ and one filter $f\;\in \;{\mathbb{R}}^{h\times d}$ (where s is the document length, d is the embedding dimension and h is the filter size), the convolution layer sequentially extracts a submatrix which has the same dimension as filter f and does the sum operation of the element‐wise product between $x\left[i:i-h+1\right]$ and f. This generates a summarised feature vector $w\;\in \;{\mathbb{R}}^{s-h+1}$ of the document matrix x by filter f with filter size h. For filter size h, multiple filters are used to capture different features.

The output vectors from the convolutional layer are then passed through an activation function such as ReLU to add non‐linearity, and a pooling layer, which extracts the maximum value of each vector. A dropout layer, which randomly sets some values in the vectors to zero, is applied to prevent over‐fitting. A final linear transformation is applied to map the vector concatenated from the pooling layer into two numeric values, representing separately whether or not the document reported the risk of bias item.

#### 2.3.2 RNN with attention

Recurrent neural network is a type of neural network which builds connections over time steps.^33^ In the hidden layer, by combining the weighted hidden representations from the previous word and the next word (if it is applied bidirectionally) through a Tanh operation, a basic recurrent neural structure can retain information in the text from both directions. RNN can handle any‐length texts and but if the sequence is very long, it is difficult to keep the information from very earlier steps to later steps because of the exploding or vanishing gradient problem.^34^ Two variants of RNNs, long short‐term memory (LSTM)^31^ and gated recurrent unit (GRU)^35^ are designed to solve this long‐term dependencies problem, which uses multiple gates (forget gate, input gate and output gate in LSTM; reset gate, update gate and output gate in GRU) for each word embedding to control the information we need to flow straight, forget, store and update to the next step.

In the general recurrent structure, the output from the hidden layer is obtained by simply taking the hidden state of the last recurrent cell, which loses some information from other recurrent cells; or averaging hidden states of all recurrent cells, which treats words at different positions equally. However, the same word may play a different role in the decision of the classification when it occurs in different sentences or contexts. A global context matrix ($\in \;{\mathbb{R}}^{s\times h}$) is created to learn the importance of each word in the document (similar to the attention mechanism described below for HNNs). The attention module is then added to learn and emphasise the word contributions to the entire document sequence.^36^

#### 2.3.3 Hierarchical attention network

Words contribute differently to an individual sentence and sentences contribute differently to the whole document. Hierarchical attention network is proposed to imitate this hierarchical structure of documents, having two levels of attention modules applied at word‐level and sentence‐level.^32^ After the recurrent hidden layer, in the word‐level attention module, the hidden representations of each word in a sentence are multiplied by a local word context vector, which is trained to learn the importance of each word in the sentence. The representation vector of each sentence is then summarised from those weighted word representations. Similarly, in sentence‐level attention, the hidden representations of each sentence in the document are multiplied by a global sentence context vector, which is trained to learn the importance of each sentence in the document. Then a document representation vector is obtained from those new weighted sentence representations. After an activation function and a linear transformation, we then output the probability for risk of bias items. With the hierarchical structure, HAN can generate ranking scores for sentences, which can be used to extract the most relevant sentences and provided to users to allow them to make a judgement on the veracity of the machine decision.

### 2.4 BERT models

One limitation of word embeddings like word2vec is that the representation vector of a given word is fixed and independent, regardless of context. Contextualised representation models like BERT^22^ (Bidirectional Encoder Representations from Transformers) address this issue. BERT extracts the contextualised embeddings by training a deep bidirectional encoder from transformers^37^ on the BooksCorpus and English Wikipedia. The transformer structure mainly consists of identical blocks, and each block contains sub‐modules based on multi‐head self‐attention and a feed‐forward neural network. It dispenses with recurrence and convolutions, and achieves state‐of‐the‐art performance on many NLP tasks.^37^ The pre‐trained BERT can be fine‐tuned with a simple additional output layer for downstream tasks. BERT uses WordPiece with a 30,000 token vocabulary for tokenization, which handles rare words better than the ‘pure’ word embeddings and more efficiently than character embeddings.^38^

Previous work shows that the domain corpus used for pre‐training affects the performance of the downstream task.^39^ Since our task is conducted on preclinical texts, we use the pre‐trained weights from BioBERT to initialize the model, which applies the same architecture as BERT and is pre‐trained on combinations of text corpora including BookCorpus, English Wikipedia, PubMed abstracts and PubMed Central full‐text articles.^40^

One drawback of BERT is that it can only accept embeddings of maximum 512 tokens as input, which limits the usage for tasks with long documents. There are other transformer models designed for long documents, such as Longformer^41^ which can process a maximum of 4096 tokens. However, this is still computationally expensive, and our full‐text publications contain 5000 tokens on average. To solve this issue, we propose two strategies.

#### 2.4.1 BERT with document chunk pooling

We split documents into text chunks, apply BioBERT to each chunk, and pool the hidden states from different chunks using multiple strategies. This is similar to the structure applied in the classification of clinical notes for patient smoking status,^42^ with some modifications as shown in Figure S4. After the WordPiece tokenization, considering a document with s tokens, the document is split into m = [s/510] chunks (excluding the first token indicating classification and the separation token for sentence segmentation). The input representation of the document is $X\;\in \;{\mathbb{R}}^{m\times 512\times h}$, where h is the hidden dimension throughout the embedding layer and encoder layers in BioBERT. Instead of taking the hidden states from the last encoder layer, we perform the average pooling operation over several encoder layers to obtain the output. We summarise across tokens within each chunk with five different options: (1) max pooling, (2) average pooling, (3) concatenate output from max pooling and average pooling, (4) use hidden states of the first token, (5) concatenate hidden states of all tokens. After two pooling layers, we explore three head layers (linear, convolution or LSTM) for the downstream classification task. The convolution and LSTM heads use the same architecture as described previously. The linear head cannot handle sequences of different lengths, so we add another pooling layer to obtain the fixed‐dimension output. The pooling methods use the same options applied in the second pooling layer, with the exclusion of ‘concatenate hidden states of all tokens’, which does not generate a fixed‐dimension output.

#### 2.4.2 BERT with sentence extraction

Instead of using the full‐text document as input, we extract the most relevant sentences to the risk of bias description. We first use scispaCy^43^ to split a document into sentences, and then apply SentenceTransformers^44^ to obtain a vector for each individual sentence. We also feed a description sentence of each risk of bias item (see descriptions in 2.1 Dataset) to the Sentence‐BERT^44^ and obtain the corresponding representative vectors. For each individual document, we calculate the cosine similarity score between each sentence vector and the vector of the risk of bias description sentence. We take the first *k* sentences with the highest similarity scores, that is, the most *k* relevant sentences, to form a new shorter passage. We then fine‐tune the DistilBERT^45^ model (a lighter version of BERT), with a linear, convolution or LSTM head on the new passage, to generate the probabilities of risk of bias reporting. The sentence extraction process is unsupervised and is independent of the actual training process.

### 2.5 Evaluation metrics

To evaluate model performance, we define ‘True Positive’ as the number of records which report the risk of bias item and are predicted as reported; ‘True Negative’ as the number of records which do not report the risk of bias item and are predicted as unreported; ‘False Positive’ as the number of records which do not report the risk of bias item but are predicted as reported; and ‘False Negative’ as the number of records which report the risk of bias item but are predicted as unreported. For all classification models described above, we calculate four metrics of performance, being (1) **Recall** (or Sensitivity) = True Positive/(True Positive + False Negative); (2) **Precision** = True Positive/(True Positive + False Positive); (3) **F1 score** = (2 × Recall × Precision)/(Recall + Precision); and (4) **Specificity** = True Negative/(True Negative + False Positive). Recall measures the portion of records which are identified as reported among all truly reported records. Precision measures the portion of truly reported records among all records identified reported. Specificity measures the portion of records identified as unreported among all unreported records. F1 is the harmonic mean of recall and precision, and we use this as the main metric for hyperparameter and model selection.

## 3 RESULTS

The performance of eight models from three categories (baselines, neural models and models using BERT with two strategies) on the validation set are shown in Table 3. For baseline models, all items achieve F1 score over 48% and particularly, models for compliance with animal welfare regulations show good performance, with F1 around 90%. For the selection of text representation methods, from our experiments, bag‐of‐words is not robust and prone to be over‐fitting. Doc2vec gives the best results across all items, perhaps because the training sample texts for doc2vec are closer to the preclinical domain, while the pre‐trained word2vec vectors are induced from the more general biomedical corpus. For model selection, logistic regression achieves the best performance for random allocation to treatment or control, blinded assessment of outcome and conflict of interests; while for compliance with animal welfare regulations and animal exclusions, support vector machine performs better.

**TABLE 3:** Performance of best model in three categories (baseline, neural model and BERT models with two strategies) for risk of bias items on the validation set

Risks of bias item	Model	F1	Recall	Precision	Specificity
Random allocation	SVM	51.9	72.2	40.5	65.1
	LogReg	56.3	75.3	44.9	69.7
	RF	67.2	79.9	58.1	81.0
	CNN	86.4	93.2	81.8	92.8
	**RNN + Attn**	**87.2**	92.4	83.7	93.7
	HAN	86.2	91.3	83.1	93.7
	BERT‐DCP	85.4	92.7	80.1	92.2
	BERT‐SE	80.6	82.0	82.0	92.7
Blinded assessment of outcome	SVM	59.3	67.8	52.7	74.7
	LogReg	60.0	69.1	53.0	74.6
	RF	57.8	68.3	50.2	71.8
	CNN	82.4	88.5	77.8	89.4
	RNN + Attn	83.0	91.1	77.2	88.5
	HAN	81.3	86.4	77.5	89.1
	**BERT‐DCP**	**83.1**	91.8	77.0	87.7
	BERT‐SE	79.9	84.7	79.8	89.9
Conflict of interests	SVM	67.1	79.7	57.9	77.7
	LogReg	68.8	76.1	62.8	82.6
	RF	65.1	68.5	61.9	83.8
	**CNN**	**84.5**	86.8	84.1	93.8
	RNN + Attn	82.9	85.4	82.0	92.9
	HAN	83.2	84.7	82.8	93.2
	BERT‐DCP	79.5	84.6	76.8	90.1
	BERT‐SE	64.0	64.3	70.9	88.3
Compliance of animal welfare regulations	SVM	90.1	96.3	84.6	42.8
	LogReg	87.6	85.4	89.9	68.7
	RF	88.8	89.7	88.0	60.2
	CNN	86.9	83.3	92.4	97.4
	RNN + Attn	76.3	77.6	78.3	93.5
	HAN	79.3	77.9	84.5	94.9
	BERT‐DCP	93.8	92.1	95.8	87.7
	**BERT‐SE**	**94.0**	94.6	93.8	75.1
Animal exclusions	SVM	39.0	64.3	28.0	72.5
	LogReg	41.4	62.5	31.0	76.8
	RF	48.8	44.6	53.8	93.6
	**CNN**	**60.2**	73.6	54.2	89.7
	RNN + Attn	58.0	68.3	54.3	90.0
	HAN	53.4	58.4	54.0	88.9
	BERT‐DCP	56.2	77.0	46.8	84.7
	BERT‐SE	34.4	46,5	30.5	79.5

*Note*: ‘SVM’ represents support vector machine; ‘LogReg’ represents logistic regression; ‘RF’ represents random forest; ‘CNN’ represents convolutional neural network; ‘RNN + Attn’ represents recurrent neural network with attention; ‘HAN’ represents hierarchical attention network; ‘BERT‐DCP’ represents BERT model with document chunk pooling; ‘BERT‐SE’ represents BERT model with sentence extraction. For each risk of bias item the best performing approach (by F1 score) is given in bold.

We find that neural models are more robust to hyperparameter tuning than baseline models. For random allocation, blinded assessment of outcome and conflict of interests, neural models improve F1 by 14%–30% over baseline models, with little difference among the three neural models. For compliance with animal welfare regulations, neural models have no advantage over baselines, with F1 falling 4%–14%. For animal exclusions, weight balancing strategy and under‐sampling do not effectively address the data imbalance issue, and the training process is prone to over‐fitting.

Models using BERT with the two strategies described do not outperform neural models, except the item of compliance of animal welfare regulations, which has 3% improvement. This is expected because in the document chunk pooling (DCP) strategy, we do not take any advantages of BERT structure by freezing all the encoder layers, and multiple pooling strategies do not address this limitation; in the sentence extraction strategy, although we can fine‐tune DistilBERT, we still lose some information by using shorter texts extracted from full publications. We have not been able to evaluate the performance of sentence extraction modules, which requires further sentence‐level annotations.

Using the best model in its optimal setting for four risk of bias items, we evaluate and compare the performance with our previous regular expression approach on the test set (no regular expression approach has been developed for animal exclusions). For blinded assessment of outcome, we select the RNN with attention as the optimal model instead of BERT with document chunk pooling (BERT‐DCP) because we do not consider the very small advantage to justify the complexity of pre‐processing in the latter approach. Table 4 shows our models improve performance by between 13% and 36% for four risk of bias items compared with regular expressions (each significant at *p* < 0.05 by the McNemar's test^46^).

**TABLE 4:** Performance of the best natural language processing model and regular expression approach for each risk of bias item on the test set

Risks of bias item	Model/approach	F1	Recall	Precision	Specificity
Random allocation	RNN + Attn	**82.0** *	86.8	79.5	89.7
	Regular expression	68.8	96.4	53.6	62.7
Blinded assessment of outcome	RNN + Attn	**81.6** *	87.8	78.2	88.4
	Regular expression	68.3	59.8	79.6	92.1
Conflict of interests	CNN	**82.7** *	80.6	86.2	93.9
	Regular expression	48.7	33.8	87.1	97.8
Compliance with animal welfare regulation	BERT‐SE	**91.5** *	91.4	92.0	70.9
	Regular expression	55.2	40.9	85.2	78.2
Animal exclusions	CNN	46.6	56.5	45.0	89.7
	Regular expression	–	–	–	–

*Note*: A regular expression approach has not been developed for animal exclusions. ‘CNN’ represents convolutional neural network; ‘RNN + Attn’ represents recurrent neural network with attention; ‘BERT‐SE’ represents BERT model with sentence extraction.

* *p* < 0.05 v Regular Expression approach, McNemar's test.

Table 5 shows the prediction and sentence extraction function of our models on an example paper which reports random allocation, blinded assessment of outcome and animal exclusions, but does not report conflict of interest and animal welfare regulations. Unlike previous rule‐based approaches which output yes/no labels only, our models can be used to extract the most relevant sentences from full text, which can enhance the judgement from the prediction probabilities, or provide signals whether users need to re‐check the full texts. In Table 5, sentences extracted for random allocation, blinded assessment of outcome and animal exclusions indicate the clear relation with the items and positive evidence for the prediction probabilities, while sentences extracted for conflict of interests and animal welfare regulations bearing little relation to the target concept, which proves the predictions in a different direction.

**TABLE 5:** An example of model predication and relevant sentence extraction for risk of bias items on a full‐text publication

Risk of bias item	True	Prediction	High‐scored sentences
Random allocation	Yes	99.97%	In the last 5 min of this habituation period, three 5 s, 56 dB, substartle‐threshold white noise tones were presented randomly by computer
Blinded assessment of outcome	Yes	99.99%	Video records of 11 randomly selected animals were recorded by an observer blind to the experimental conditions
Conflict of interests	No	0.32%	Schematic depictions of the regions dissected for neurochemical analysis are presented in Figure 2
Animal welfare regulations	No	3.68%	Role of the amygdala in the coordination of behavioural, neuroendocrine and prefrontal cortical monoamine responses to psychological stress in the rat
Animal exclusions	Yes	99.99%	In 8 of the original 26 lesioned animals in the pre‐training experiment, the lesions were judged incomplete by the criteria above and were excluded from the data analyses

*Note*: Prediction probabilities are generated from the optimal model of each item, and most relevant sentences are extracted by the hierarchical attention network.

## 4 DISCUSSION

**FIGURE 2:**
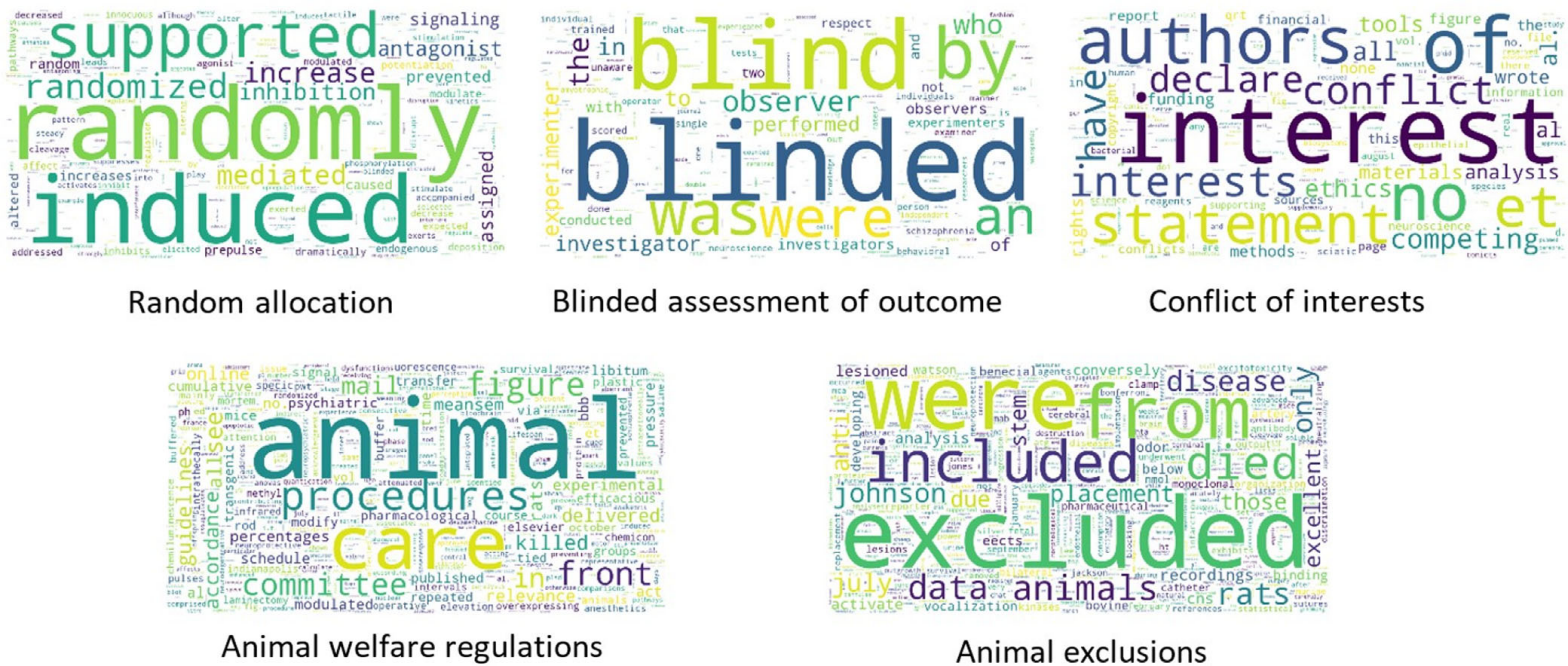
Most important words in the decision of classification for each risk of bias item, based on the average attention scores from RNN output over all positive samples [Colour figure can be viewed at wileyonlinelibrary.com]

We have shown that different models are optimal for the detection of reporting of different risks of bias. Convolutional neural network is best for conflict of interests and RNN with attention are best for random allocation and blinded assessment of outcome. For compliance with animal welfare regulations, models using BERT with sentence extraction (BERT‐SE) strategy achieve the best performance. For animal exclusions, CNN achieves the best performance on the validation set, but no approach provides reliable performance on the test set. Compared with the previous regular expression approach, the F1 scores for four risk of bias items are between 13% and 36% higher, indicating a substantial improvement. The sentence extraction function can provide potentially relevant sentences as clues for users making the judgement. We can analyse all positive samples and use the RNN with the attention module to output attention scores for tokens in each individual paper, thus we can extract the most important words in the decision of classification task (Figure 2), which may help the development of future rule‐based approaches.

**FIGURE 3:**
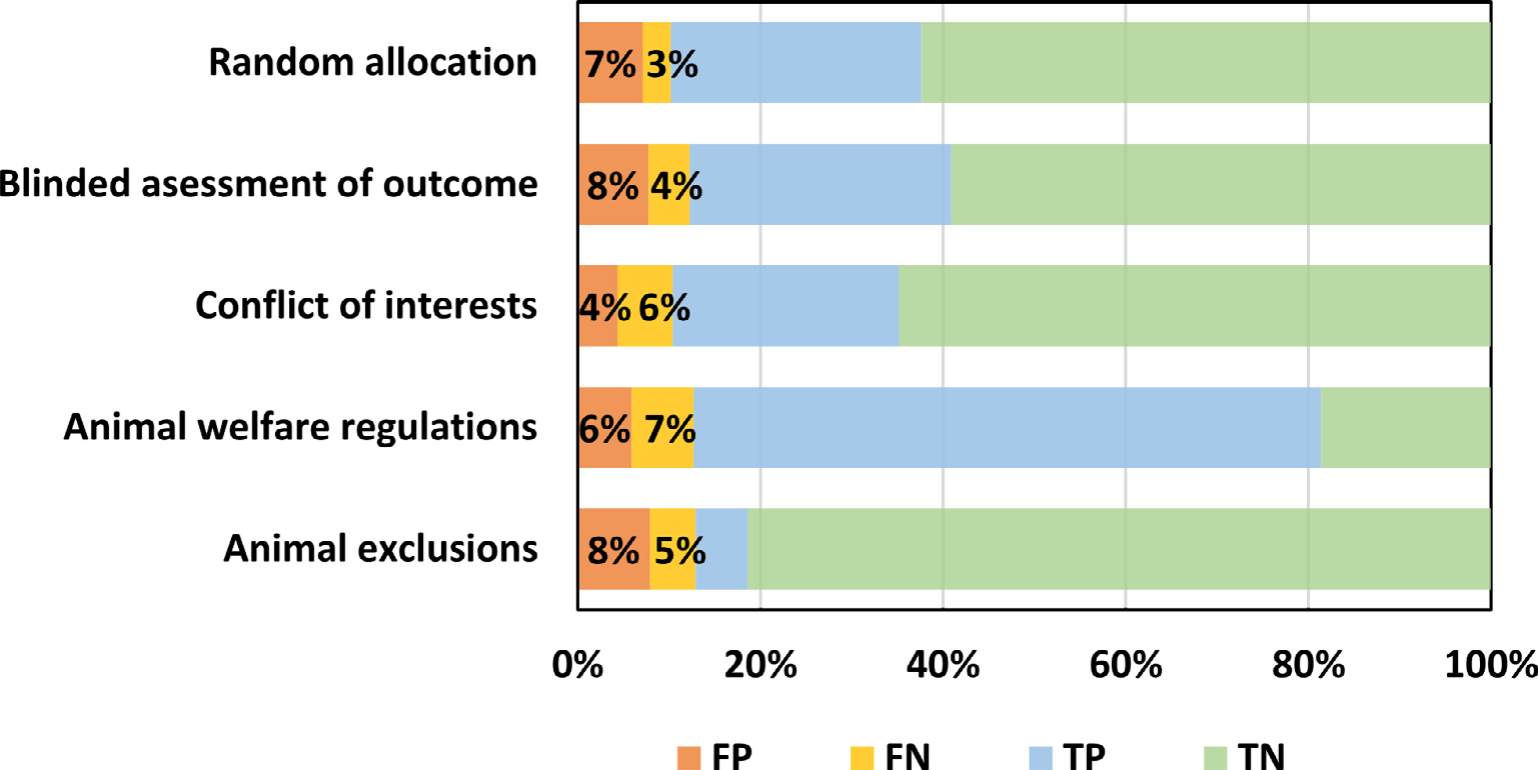
Percentages of false positive, false negative, true positive and true negative of each optimal model for the corresponding risk of bias item on the test set [Colour figure can be viewed at wileyonlinelibrary.com]

Among the incorrect records, our models are more likely to conclude that papers report random allocation, blinded assessment of outcome and animal exclusions, and less likely to predict that papers report conflict of interests and animal welfare regulation (Figure 3). To analyse sources of error, we randomly selected 10 incorrect records for each item from the test set. Our models did not recognise phrases like ‘unaware’ for blinded assessment but considered that ‘animals are randomly selected for testing’ indicated random allocation to the experimental group. It may be that most records in our training set describe random allocation based on the presence of the word ‘random’ and blinded assessment based on the word ‘blind’, and that our training corpus did not have sufficient examples of alternative valid descriptions for these to be learned. We also found two records where a conflict of interests was given before the ‘Introduction’ section or after the ‘Reference’ section, where we had removed the relevant text at the text processing stage.

The tool and codes for predicting probabilities of risk of bias reporting in preclinical full texts is available at https://github.com/qianyingw/pre-rob. The levels of performance achieved make these tools suitable for research improvement activity where several hundred publications are to be evaluated. For instance, for random allocation in a corpus of 1000 manuscripts, this approach would estimate prevalence within 3% of the true value and for 100 publications, within 10% of the true value (see calculations at https://github.com/camaradesuk/confidence_intervals_simulation). Given that the changes sought in research improvement activities are at least of this magnitude, we consider the performance of these tools in determining the reporting of risk of bias items to be such that they are suitable for deployment in a research improvement context. Similarly, they are suitable for the evaluation of risk of bias in large corpuses such as large preclinical systematic reviews. However, they are not yet at the level where they are appropriate for the evaluation of individual publications.

Our work has several limitations. First, our training dataset includes publications drawn from three datasets focusing largely on the neurosciences, as well as two datasets from unselected preclinical studies published in *PLoS One* and *Nature*. This may influence the generalizability of our findings. Second, PDF to text conversion loses document structure and we cannot identify the main sections of publications. This introduces some noise (for instance text from figures and tables) to our training corpus. Tools like GROBID (https://github.com/kermitt2/grobid) can convert PDFs to structured XML but it highly depends on the quality of PDF, and in our experience it does not work well for some preclinical publications. However, enhanced approaches to PDF conversion, and increased availability of publications in XML format, means that this approach may become feasible in the future. Finally, following our own systematic review practice, we considered all publications which did not report measures to reduce risks of bias to be at high risk of bias, with no ‘unclear’ category. Because our models output continuous probability scores ranging from 0 to 1 which can be then used to provide a binary score, it might be possible to identify the ‘unclear’ category as those with intermediate scores; but since these are not labelled in each of our datasets we have not been able to do so.

In future work, we will seek to improve performance further, using datasets involving more journals and a wider range of preclinical experiments, and will exploit diseases and texts from structured PubMed XMLs, which may yield better performance. We will continue improving the attribution of animal exclusions to achieve more reliable performance and we will develop approaches for other risk of bias items including sample size calculation and allocation concealment. We will also develop a user‐friendly function embedded in the preclinical systematic review facility SyRF^47^ and a standalone API, enabling usage to others.^48^

## 5 CONCLUSIONS

We explore multiple text classification models, from baselines to recent NLP techniques and demonstrate the advantages of neural models and BERT models for risk of bias assessment in preclinical literature. BERT models work well for animal welfare regulations, while convolutional or RNNs achieve better performance for. We encourage the use of NLP techniques to assist risk of bias assessment and reduce workflow in preclinical systematic reviews. If computational limitations require the implementation of a single tool, we recommend using CNNs, which achieve overall good performance across our five risk of bias items. The performance of these tools is such that they could be deployed in automated approaches to monitor risks of bias reporting as part of institutional research improvement activities.

## CONFLICT OF INTEREST

The authors declare that they have no conflict of interests.

## AUTHOR CONTRIBUTIONS

Qianying Wang and Malcolm Macleod conceived the study. Mirella Lapata was involved in the design of model architecture. Qianying Wang and Jing Liao processed the full‐text data. Qianying Wang implemented the classification models, analysed results and wrote the initial draft of the manuscript. All authors provided comments on preliminary versions and approved the final manuscript.

## Supporting information


**Appendix** S1: Supporting information

## Data Availability

The data that support the findings of this study are available in the Preclinical RoB Assessment repository (DOI: 10.17605/OSF.IO/FJWX6).
